# Statistical learning of mobility patterns from long-term monitoring of locomotor behaviour with body-worn sensors

**DOI:** 10.1038/s41598-018-25523-4

**Published:** 2018-05-04

**Authors:** Sayantan Ghosh, Tim Fleiner, Eleftheria Giannouli, Uwe Jaekel, Sabato Mellone, Peter Häussermann, Wiebren Zijlstra

**Affiliations:** 1 German Sport University Cologne, Institute of Movement and Sport Gerontology, Am Sportpark Müngersdorf 6, Cologne, 50933 Germany; 2 University of Applied Sciences Koblenz RheinAhrCampus, Faculty of Mathematics and Technology, Joesph-Rovan-Allee 2, Remagen, 53424 Germany; 3 LVR Hospital Cologne, Academic Teaching Hospital of the University of Cologne, Department of Geriatric Psychiatry, Wilhelm-Griesinger Strasse 23, Cologne, 51109 Germany; 4 University of Bologna, Dept. of Electrical, Electronic, and Information Engineering, Viale Risorgimento 2, Bologna, 40136 Italy

## Abstract

Long term monitoring of locomotor behaviour in humans using body-worn sensors can provide insight into the dynamical structure of locomotion, which can be used for quantitative, predictive and classification analyses in a biomedical context. A frequently used approach to study daily life locomotor behaviour in different population groups involves categorisation of locomotion into various states as a basis for subsequent analyses of differences in locomotor behaviour. In this work, we use such a categorisation to develop two feature sets, namely state probability and transition rates between states, and use supervised classification techniques to demonstrate differences in locomotor behaviour. We use this to study the influence of various states in differentiating between older adults with and without dementia. We further assess the contribution of each state and transition and identify the states most influential in maximising the classification accuracy between the two groups. The methods developed here are general and can be applied to areas dealing with categorical time series.

## Introduction

Complex non-linear dynamical systems in nature can often be modelled to have latent discrete states^1^, and are investigated in diverse areas such as finance, medicine, robotics, and text analysis. Inference of the latent states and their causal interactions is an important aspect of such modelling where, the interplay between the various latent states can provide important insights for system characterisation and modelling. The role played by the individual latent states in the model can also be analysed for developing a parsimonious description of the system^2^. Human locomotion is a complex dynamical system and various aspects of locomotor behaviour have been studied, for example in the distinction between normal and pathological gait^3^, analyses of gait and postural stability^4–9^, assessment of fall-risk^10–12^, in mobility studies^13–15^, in the progression of dementia^16^, and more recently in evaluating the cognitive impairment in older adults^17^.

The majority of these recent studies have concentrated on feature set generation with respect to controlled locomotion tasks or motor states and validated the algorithms; thus were limited to a very narrow range of locomotor behaviour. In free living conditions, where a multitude of activities are performed (which could lead to a large number of underlying states), not only the classification of the typical states, but the sequences of transitions from one state to the other can provide useful insight into the dynamics of locomotor behaviour. However, research effort in this context is usually concentrated on recognising physical activities^18–21^ (also called as Human Activity Recognition or HAR). Some effort has been made in understanding the temporal evolution of the activity in humans through actigraphy, especially in the context of circadian rhythms by investigating the two state (active/inactive) models^22,23^, which can overlook certain temporal variations in the locomotor behaviour that might be characteristic of certain population groups^13^.

In this work, we develop a general method to address the latter issue through statistical learning approaches, and use the method to differentiate between two subject groups based on their locomotor behaviour. We study the locomotor behaviour through long-term body-worn sensor measurements and categorise them into different locomotor states (hereafter “states”). We then study these individual states by defining the probability of occurrence, designated as State Probability (SP) and the Transition Rates (TR) between the different states. As will be seen in the ensuing, the construction of these two feature sets is completely general over the dimensions (number of states), and observation time and can be adapted to study temporal variations in locomotor behaviour.

As a proof-of-concept demonstration, we study the differences in the locomotor behaviour between institutionalised patients suffering from dementia and healthy community-dwelling older adults using a variety of supervised classification algorithms. Neuro-degenerative diseases such as dementia manifest as wide ranging impairments in psycho-social and locomotor behaviour^24^. Sensor based evaluation of locomotor behaviour can be used to objectively quantify aberrations and impairments for quantitative assessment, online diagnosis, and development of targeted therapeutic protocols^13,25^. We identify the activity states and the transitions relevant for the classification of the two groups. The simplicity of the SP and TR methods lend to a wide generalisability of the features for application in many real-life scenarios, where a long term monitoring of the subjects is required. We show that the TR method outperforms SP method in classification tasks, thereby suggesting that the manifest dynamics underlying the structure of long-term locomotor behaviour can be instrumental in understanding the daily activities of subjects suffering from various mobility impairing diseases.

## Results

We have derived a seven state representation^26^ of the locomotor behaviour for our analysis, namely: Lying (Sup), Sitting Sedentary (SiSe), Standing Sedentary (StSe), Postural Transitions (PoTr), Sitting Active (SiAc), Standing Active (StAc), and Gait (see Methods). In the following, we initially sketch the statistical and distributional properties of the seven state probability (SP) features, and then apply the SP, and the associated TR features for statistical learning. Two exemplary groups of subjects have been studied here: community living older adults, and institutionalised patients suffering from dementia.

### Summary statistics of features

**Figure 1:**
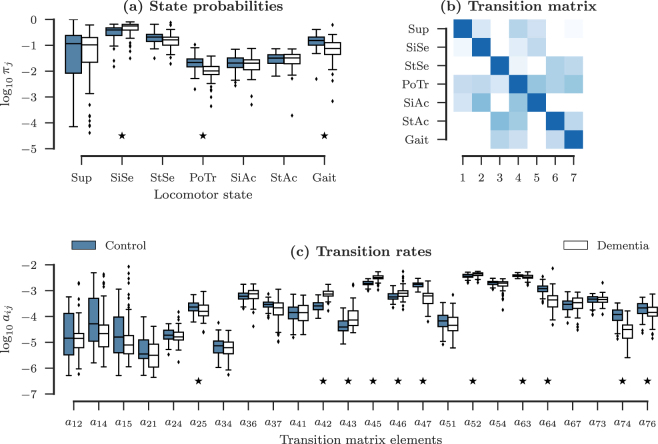
Summary statistics of features. The summary statistics of the two feature sets SP and TR are shown in this figure. Panels (a and c) show the box plots for SP and TR respectively for the two groups (control in dark, and dementia in white). The states and transitions at which the two groups differ significantly, calculated through the Mann-Whitney U-test (*p* < 0.001), have been highlighted using black stars. The panel (b) displays an empirically constructed transition matrix representative of the control group subjects. The dark pixels represent higher transition rates, the shade lowering with decreasing transition rate. The null transitions are shown in white. The *y*-axis of the transition matrix represents the numerical coding of the seven states for clarity in interpreting the transition matrix elements (Sup corresponds to state 1, and Gait to state 7). In the panel (c), diagonal elements and the null transition elements have been dropped to preserve visual clarity. Also, all quantities have been plotted on a logarithmic (base-10) scale to highlight the distributional variations amongst the groups.

We have represented the summary statistics and the distributional characteristics of SP of the two groups in Table 1. We note here that as shown in the table with *, all relevant parameters such as skewness, kurtosis, results of the Shapiro-Francia test and the Mann-Whitney U test are deemed significant only at the *p* < 0.001 level, unless stated otherwise. We observe that a subset of states in the control group exhibit significant skewness and kurtosis (Sup, StSe, PoTr and SiAc); while the dementia group show significant skewness for Sup, StSe, SiAc, and Gait. The other states show very weak skewness. The distributional characteristics of the SP is ascertained through the Shpario-Francia test, which rejects the null hypothesis of normal distribution in concordance with the results obtained for significant skewness and kurtosis. The *p*-values are reported in Table 1. The differences in the distributions of the two population groups are reported through the the Mann-Whitney U test, and we see that the two population groups are significantly distinguished from each other for SiSe, PoTr, and Gait. We have also represented these results graphically in Fig. 1 (panel a), where the SP have been plotted on a log 10 scale for better visualisation. We also observe that StSe shows distributional difference between the two groups at *p* < 0.001 significance level.

**Table 1:** Summary statistics of the probability of physical activity for the control and dementia groups.

Parameters	State probabilities for locomotor behaviour						
	Lying (Sup)	Sitting Sedentary (SiSe)	Standing Sedentary (StSe)	Postural Transition (PoTr)	Sitting Active (SiAc)	Standing Active (StAc)	Walking (Gait)
Mean^¶^	18.8	35.5	21.4	2.59	2.46	3.41	15.9
	14.1	51.2	18.4	1.21	2.25	3.41	9.42
Standard Deviation^¶^	23.5	15.2	10.6	1.84	1.58	1.76	8.25
	15.3	19.3	13.2	0.732	1.43	1.72	8.49
Median^¶^	11.6	39.2	20.8	2.17	2.06	3.20	15.3
	10.4	56.1	16.3	1.03	2.06	3.35	7.45
25^*th*^ percentile^¶^	0.846	24.1	14.1	1.46	1.38	2.01	10.2
	2.16	39.1	9.74	0.741	1.18	1.97	4.41
75^*th*^ percentile^¶^	24.0	47.3	26.6	2.96	3.25	4.32	20.9
	19.0	64.9	20.7	1.52	2.78	4.45	12.8
Skewness^†^	1.91*	−4.90 × 10^−1^	1.14	1.94*	9.48 × 10^−1^	4.43 × 10^−1^	4.36 × 10^−1^
	1.62*	−7.78 × 10^−1^	2.10*	1.10	1.43*	2.44 × 10^−1^	3.50*
Kurtosis^†^	3.46	−2.04 × 10^−1^	3.66	4.83	3.61 × 10^−1^	−3.90 × 10^−1^	3.79 × 10^−1^
	2.32	−2.00 × 10^−1^	5.51	1.43	2.46	−7.31 × 10^−1^	1.83 × 10^+1^
SF test^§^ (*p*-value)	1.96 × 10^−9^*	5.46 × 10^−2^	3.98 × 10^−4^*	1.34 × 10^−7^*	4.13 × 10^−4^*	4.75 × 10^−2^	2.46 × 10^−1^
	4.53 × 10^−8^*	9.23 × 10^−4^*	3.13 × 10^−8^*	6.63 × 10^−4^	1.45 × 10^−5^*	2.00 × 10^−1^	2.20 × 10^−10^*
MannU^‡^ (*p*-value)	2.38 × 10^−1^	1.22 × 10^−7^*	4.15 × 10^−3^	6.90 × 10^−10^*	2.59 × 10^−1^	4.74 × 10^−1^	1.19 × 10^−7^*

The first four moments (mean, standard deviation, skewness, and kurtosis), the three quartiles (first, median and third), and tests for normality (Shapiro-Francia), and the Mann-Whitney U test for similarity of distribution are shown. The cells with asterisks show significant behaviour at the *p* < 0.001. Refer to the table notes and the text for further discussion. The top and bottom rows for each parameter represent the statistics for control and dementia subjects respectively.

^¶^These rows shows the value of the state probabilities (*π*_*j*_ × 100) for clearer interpretation.

^†^The significant skewness and kurtosis are marked with asterisks, following the discussion in Cramer^43^.

^§^The *p*–values for the Shapiro-Francia test are shown. The *p*–values marked with asterisks show significant difference between the two groups at 99.9% confidence level (*p* < 0.001).

^‡^The *p*–values for the Mann-Whitney U test are shown. The *p*–values marked with asterisks (*) show significant difference between the two groups at 99.9% confidence level (*p* < 0.001).

We further note that, as would be expected for the older population, the mean probability of sedentary behaviour (combination of Sup, SiSe and StSe) is higher as compared to active behaviour during the observation period, with the control and the dementia subjects exhibiting mean probabilities of 0.757 ± 0.059 and 0.837 ± 0.058 respectively. The dementia group thus exhibits a higher probability of sedentary behaviour than the control group, with a higher probability of being in sedentary sitting than of lying or standing. Further, for the active physical activities, the mean probabilities of postural transitions (0.259 ± 0.002 versus 0.121 ± 0.001) and walking (0.159 ± 0.01 against 0.01) are higher in the control group than in the dementia group. Note that all the quantities mentioned above are in the form of mean ± SEM where SEM represents the standard error of mean.

Panel b of Fig. 1 shows a typical TR matrix A (see Methods). The elements with zero probability of transition have been represented by white pixels. Since transitions between some states cannot be instantaneous; for example between Sup and Gait without transitions through intermediate states such as PoTr, SiSe, SiAc, PoTr, StSe, and StAc; some of these state transitions are null, and have been excluded from the analysis. The TR matrices have been calculated as one step transition between the time steps *t*_*k*_ and *t*_*k*+1_. Note that while the TR matrix is not symmetric, the null transitions are symmetric. The time window for a typical transition between two states is of the order of hundreds of milliseconds, while the data has been acquired at a temporal resolution of 10 ms, and thus, expectedly, the within-state transition (also called residence), are rather high at the one-step transition rates as shown by the higher values of the diagonal elements of the TR matrix. The non-null TR matrix elements for the two groups are also shown in the panel c of Fig. 1. We find that transitions arising from the state PoTr to other states show distributional differences at the *p* < 0.001 significance level (denoted by black stars), with the exception of the transition from PoTr to Sup. The transitions from SiSe to SiAc, StAc to StSe, and StAc to Gait also show significant distributional differences. Furthermore, we observe that the variance and outliers of the control group are smaller than for the dementia group.

The variations in the distributional characteristics, as well as the capture of null-transitions (or physically improbable transitions) thus inherently represent the dynamical traits of locomotor behaviour. We emphasise here that the dynamics of SP and TR for different states and groups can be have diverse intrinsic representations (which we refer to as structural information), and might in principle be represented by different dynamical systems.

Despite highlighting the differences between the two representative groups, the above statistical analysis cannot however be used as a tool in a potentially diagnostic context where an online categorisation of individual subjects is envisaged. The large number of descriptive parameters in SP and TR further pose a challenge in extracting the states or transitions that are instrumental in the differing locomotor behaviour in the different groups of populations. These objectives can be achieved by statistical learning methods, which can be used to learn the relationships between the different features (the SP and TR are now considered as predictors or features), and extract those relevant to the discrimination between the groups.

### Supervised learning, classification and feature importance

#### Classification performance

**Figure 2:**
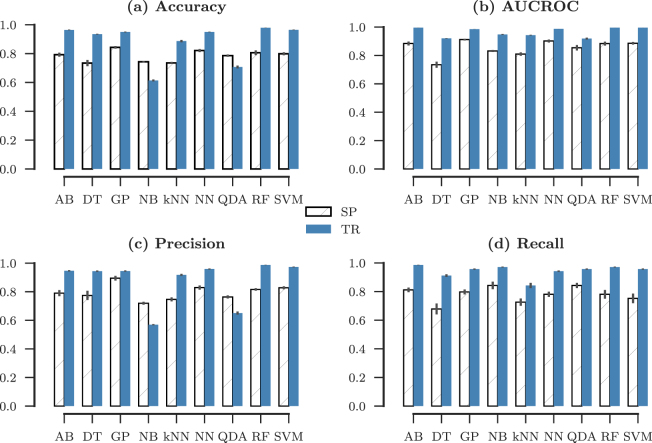
Learning performance. The classification (**a**) accuracy, (**b**) area-under-receiver-operating-characteristic-curve (AUCROC), (**c**) precision, and (**d**) recall scores (mean of 10-fold CV) for the different supervised learning methods applied to the SP (white hatched) and the TR (gray) feature sets. The errorbars represent the standard deviation of the cross validation. We observe that the classification accuracy is significantly better for the TR feature sets, expect for in the cases of Näive Bayes’, and quadratic discriminant analysis. The dark bars represent the TR feature set, while the hatched bars represent the SP features.

We have applied a number of standard supervised learning methods on the two feature sets SP and TR for classifying the two groups with distinct locomotor behaviour (see Methods). Figure 2 represents the 10-fold cross validated results of testing the algorithms on the data with 140 samples, and SP (7 features), and TR (49 features) respectively. The *k*-fold cross validation method randomly partitions the samples into *k* subsamples of equal length, with training *k* − 1 subsamples used for training, and one subsample used for testing. This procedure is then repeated *k* times, with the condition that in every iteration, the testing subsample is varied.

The Fig. 2 shows the accuracy (panel a), and area under the receiver-operator-characteristic curve (panel b) as performance indicators for the different supervised learning methods applied for the classification task. It is immediately clear from Fig. 2a that TR features outperform SP features in terms of classification accuracy. While Gaussian Processes, henceforth GP (accuracy = 0.84 ± 0.09, AUCROC = 0.91 ± 0.08), followed by Random Forest, henceforth RF (accuracy = 0.81 ± 0.12, AUCROC = 0.88 ± 0.11) is the best performing algorithm for SP, the other methods’ performance is significantly lower. In the case of TR features, with the exception of the Quadratic Discriminant Analysis (QDA), and Naïve Bayes (NB) algorithms, the algorithms have a high accuracy score of above 0.95, with the RF performing the best (accuracy = 0.99 ± 0.03, AUCROC = 1.00 ± 0.01), followed closely by AdaBoost (AB), Support Vector Machines (SVM) and Neural Networks (NN) at ≈0.95. Note that all the figures in the brackets here are CV-mean ± CV-s.d.

The precision and recall have also been shown in the panels c and d of the Fig. 2 as added performance indicators. The precision, also known as the positive predicted value, follows the accuracy trend; and the recall, also known as the specificity follows the trend of the area under the ROC curve. Specifically, the highest obtained precision for the RF method in TR is (0.99 ± 0.04), while for the GP method in SP is (0.89 ± 0.13). However, the highest recall is obtained by AB 0.81 ± 0.13 for SP, and 0.99 ± 0.04.

The performance advantage of TR over SP gives evidence that the structural information captured by TR is better for distinguishing locomotor behaviour between different groups. Further, noting the lower performance of methods involving quadratic decision surfaces, and or kernels such as QDA, and NB suggests a linear relationship between the states and behaviour.

#### Feature importance

**Figure 3:**
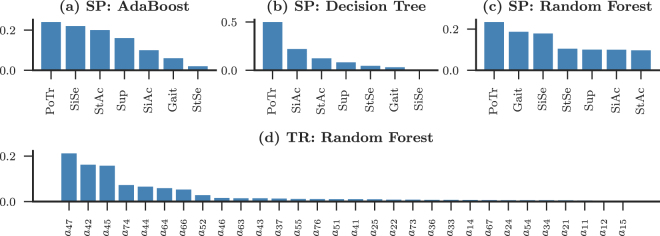
Feature importance. The importance of the features (physical activity states) calculated through the Gini impurity coefficient (*I*_*G*_) is shown in decreasing order of their importance. The panels (a–c) represent AdaBoost, Decision Tree and Random Forests respectively for the state probability features. The panel (d) represents the feature ranking for the transition rate matrix method.

The objective of this work is not only to construct a feature set that accurately distinguishes between two different population groups based on their locomotor behaviour, but also to draw quantitative insights into which states and transitions between which of these states is relevant in such classification, thus highlighting the role of specific states in the locomotor behaviour in humans. It is well known (see Methods) that many of the statistical learning methods transform the feature sets, during the process, thereby making interpretation of the selected features difficult. Thus, we have used the ensemble based methods to quantitatively analyse the importance of the states and the associated transitions in the classification task. The feature importances calculated as the Gini impurity (*I*_*G*_)^27,28^, are plotted in Fig. 3, in decreasing order of magnitude. The feature importance for SP have been shown for the three ensemble methods AB (panel a: accuracy =0.79 ± 0.12), DT (panel b: accuracy =0.73 ± 0.16) and RF (panel c: accuracy =0.81 ± 0.12); while for the TR features, only the RF method (accuracy =0.99 ± 0.03) has been represented in panel d for brevity.

In the case of SP, all the ensemble based methods (AB, DT, and RF) select postural transitions (PoTr) to be the most relevant feature facilitating the discrimination between the control and dementia subjects (represented in the panels a–c of Fig. 3). The interesting aspect of the feature importance ranking is the similarity in the importance of some features. For example, in the case of Random Forest (panel c), while PoTr, Gait and SiSe have high “relative” importance, the other four states have similarly low importance (<0.1). Since we have ${\sum }_{k}\,{I}_{G}=1$ over all the *k*–features, PoTr, Gait, and SiSe together can be interpreted to have the maximum relevance, while the rest have low and nearly equal relevance. For AdaBoost (panel a) and Decision tree (panel b), the importance ranking has a more gradual slope in comparison. However, it is clear from the three methods that PoTr is the most relevant feature in the classification task. This is also in concurrence with the observation earlier that postural transition showed significant distributional difference between the two groups. Further, we observe that Gait, SiSe and SiAc also appear as the highest ranking features in the three ensemble based methods. While the three methods are not in general agreement over the ranking of the second and third relevant features, we will see in the proceeding that they play an important role in the classification in terms of the transitions from these states.

The *I*_*G*_ for the TR features are shown in the panel d of Fig. 3. Following the discussion above, again, the transitions emitting and terminating at PoTr were selected as by the RF algorithm to have have a high relevance in the classification task, with a visual inspection of the importance ranking revealing that the transitions PoTr to Gait (*a*_47_), PoTr to SiSe (*a*_42_), and PoTr to SiAc (*a*_45_) contribute in a major way, while Gait to PoTr (*a*_74_) is also an important transition. The relative difference in the contribution of *a*_47_ and *a*_74_ might be attributed to the asymmetry in the transition rates. Reminding ourselves that the residence rate have a higher relative magnitude in the TR matrix due to the high sampling rate, we observe that *a*_44_ and *a*_66_, i.e. the residence in PoTr and StAc have an important role in discriminating between the two groups. A probable cause of the inclusion of *a*_66_ in this feature importance suggests that the control subjects are expected to be more active during the observation period which corresponds to day time locomotor behaviour.

To summarise the results, we note the following points:

1. The feature importances obtained through the ensemble based methods confirm PoTr to be the most important discriminatory feature;
2. The TR method outperforms the SP method in terms of classification accuracy, and discriminatory capabilities;
3. And, ensemble based methods, owing to their easily interpretable feature importance, allow us to draw clinically relevant conclusions about the efficacy of the methods employed in this paper.

## Discussion

In this work, we have developed a general method for studying a wide range of dynamical physical systems that can be observed or described as categorical time series. The SP and TR methods described here are generalisable to any number of dimensions and can be used to study any observation period. As a proof-of-concept application, we focussed on drawing insights into the locomotor behaviour in humans and derive the states which distinguish between groups that show distinctive behaviour. To this end, we extracted a range of core states commonly encountered in daily living conditions and derived the state probabilities and the transition rates between the underlying states. We analysed the feature sets thus obtained through conventional statistical methods, and statistical learning methods. We showed that the transitions between the states capture the rich dynamical structure of the locomotor behaviour which can be used with a high degree of accuracy to distinguish between two different groups, while automatically excluding physically unlikely transitions between states. We further identified the states and corresponding transitions that play a pivotal role in distinguishing these characteristic behaviours.

We showed that the probability of a patient suffering from dementia being in a sedentary state (83.7%) is more likely than a healthy older adult (75.7%) in our time frame, which agree with other findings^29^. We also showed that dementia subjects are less likely to be in the state of gait (9.42%) compared to the healthy older adult (15.9%)^13,25^, which can be attributed to the psycho-motor impairment in advanced dementia^30^.

We further used SP and TR methods to distinguish between the two older population groups and found that TR outperformed the SP method in classifying the two groups, showing the TR capture the dynamical structure of human locomotion more effectively than the SP, and has better predictive capabilities, where the ensemble methods outperformed the other methods, suggesting their suitability in such dimensional classification tasks, while automatically performing feature relevance. This could be of particular significance to the clinical and biomedical community, where the development of diagnostic and therapeutic protocols and interventions can be assisted by knowledge of specific states requiring attention. However, various factors that can have possibly had an impact on the performance of these methods are the different physical environmental conditions (home versus hospital), age difference, and the efficiency of the HAR algorithm. We have shown that these conditions did not have a substantial impact on the locomotion behaviour in the two population groups as evidenced by the results of conventional statistical tests, which was a further motivation to employ machine learning techniques to investigate differences in the locomotor behaviour. This is a proof-of-concept application of the methodology developed in this work, and while we have applied it for discrimination of dementia in the elderly, this method can be applied to other time dependent dynamical systems which can be described based on state changes.

We further identified that the state most likely to contribute to the differences between the healthy and patients suffering from dementia is postural transitions, which appears to be the logical intermediate stage between two different states, which were confirmed by the TR method to identify the most contributory state transitions included PoTr.

Clinical assessment protocols in dementia are often based on the observation of behavioural symptoms. Our method relies on an objective quantitative assessment of locomotor behaviour, which can be performed in a clinical context, with minimal human intervention, and without subjective interpretation. We expect that the objective assessment of behavioural states and use of machine learning techniques will become relevant to support clinical decision making in dementia.

In conclusion, we have demonstrated a method for studying dynamical systems representable by categorical time series and have used them to derive important categories contributing to the dynamics through statistical learning. This method in turn, can be used not only for online prediction affording the clinical community an unbiased and objective method for subject classification, but can also be used for quantitative studies in the temporal locomotor behaviour of subjects. This analysis could also potentially play a role in pre-clinical investigation of the motor dysfunctions associated with various pathologies.

## Methods

### Study design and participants

Subjects (*n* = 140) in two groups (control and dementia) were recruited from community living older adults^31^, and three specialised acute dementia care units of the LVR-Hospital Cologne (the randomised clinical trial was registered in the German Clinical Trial Register with reference number DRKS00006740 on October 28, 2014)^13^, respectively. Equal number of subjects in each group were studied in this investigation. The male-to-female ratio was 1:1, and 0.71:1 in the dementia (mean age = 80.93 ± 6.28 years) and control groups (mean age = 69.49 ± 4.15 years) respectively. The body mass index of the control and dementia subjects were 24.8 ± 4.1 and 24.9 ± 4.1 respectively. Nineteen control and 7 dementia subjects had a higher education, while 18 control and 3 dementia subjects finished high school as their highest level of education. Of the control subjects 20 had middle and 12 had lower education levels, in the group of dementia subjects, these figures were 43 and 8, respectively. Education data for one subject was not available. All subjects were included in the study only upon written confirmation of non-objection from their respective physicians.

The dementia subjects were evaluated by two senior geriatric psychiatrists (unrelated to this investigation) for confirmation of diagnosis Subjects with a confirmed diagnosis of dementia according to the International Classification of Diseases, version 10 (ICD-10)^32^, were included in the study. Psychiatric assessment of the dementia patients were performed with various assessment methods such as the Neuro-Psychiatric inventory (NPI = 22.4 ± 13.6), Cohen Mansfield Agitation Index (CMAI = 51.5 ± 12.5), and Mental Mini State Examination (MMSE = 17.8 ± 5.2). Forty subjects were administered only antipsychotics (2.4 ± 1.9 mg/day), one subject was administered only sedatives (3.3 mg/day), while fourteen subjects were administered both sedative (3.2 ± 1.9 mg/day) and antipsychotic (3.3 ± 1.9 mg/day) medication.

The control subjects were included in the study only if they did not exhibit any serious neurological or psychiatric symptoms, and had no diseases that could hamper mobility. While 11 subjects did not report any disease, 22 control subjects had been diagnosed with one (9 with endocrine, 7 with cardiovascular, 5 with orthopaedic and 1 with eye or ear) disease. 36 subjects exhibited more than one disease, 4 subjects had minor neurological or psychiatric symptoms, 25 had cardiovascular symptoms, 21 had symptoms of endocrine diseases, 6 showed eye or ear diseases, 18 had orthopaedic symptoms, and 8 subjects had tumours.

### Instrumentation

The uSense and Samsung SIII acted as sensing units and raw data was exported and processed through the same software for both devices. Commercial inertial measurement units (IMUs) MPU9150 and MPU6050 (TDK Invensense) are embedded in the uSense and Samsung SIII devices respectively. Both chips have equivalent range and resolution (±2 *g* for the accelerometer and ±250°/s) for the gyroscope) and have the same sampling rate (100 Hz). The equivalence of these two IMUs has been investigated^33^ and verified for the two devices.

### Data acquisition

All the subjects were monitored in their daily living conditions: acute dementia care units of psychiatric hospitals (dementia) and home living (control), without any restrictions and without imposing any standardised conditions such as in a laboratory environment. The dementia subjects were monitored continuously for at least forty-eight hours, while the control subjects were monitored over five days between waking up and sleeping. Owing to the variations of the daily sleep-wake patterns of individual control subjects, and the unavailability of night-time data, the raw data obtained from both populations were synchronised to have a duration of eight hours between 12:00 and 20:00 hours. In order to preclude effects of sample size, only one eight hour observation period from each subject was used in the study.

The sensors were placed at the lower back (approximately the fifth vertebra of the lumbar column, L5) with elastic waist bands (control^31^), and waterproof adhesive foil (dementia^13^, Opsite FlexiFix, Smith & Nephew Medical Ltd., Hull, England). The motivation for the L5 placement was the report that the optimal placement position of IMU for locomotor behaviour monitoring is the lower back or the ankle^34^. Three dimensional acceleration, and angular velocity were sampled at 100 Hz in both cases.

### Signal processing and locomotor state detection

The non-commercial signal processing and feature extraction software implemented in MATLAB (MATLAB R2015b, The MathWorks, Inc., Natick, MA) is an outcome of the FARSEEING project (grant agreement No. 288940 funded under the European Union Seventh Framework Programme (FP7/2007–2013); it allows quantitative as well as qualitative data analysis and it has been validated to identifiy locomotion behaviour inpatients with dementia^13^, in older adults residing in independent-living retirement homes^35^, and in community-dwelling older adults^36^.

The software has been further validated within the scope of the PreventIT project (grant No. 689238 funded under the European Union Horizon 2020 program H2020-EU.3.1), where it was tested on two datasets of elderly subjects: 1) the ADAPT dataset^26^, where video recording was performed using ceiling mounted cameras in lab settings and an action camera in free-living conditions; and 2) a dataset from the University of Auckland^35^ where subjects performed both scripted and unscripted activities of daily living collected in a free-living environment. Making use of frame-by-frame video annotations as gold standard, the accuracy of the walking intervals detection is ≥90% in both datasets.

An interval is labelled as “sedentary” if associated Metabolic Equivalents (METs) are below or equal to 1.5^37^, otherwise the interval is labelled as “active”. METs estimate method is in agreement with Sasaki *et al.*^38^. Detection of postural transitions is based on the trunk acceleration and orientation^39^. “Sedentary” intervals with a mean angle between the vertical axis and the medio-lateral or the anterior-posterior direction of the trunk below 30° are labelled as “lying”; the distinction between “sitting” and “standing” states is based on the identification of walking bouts preceding/following a postural transition. “Active” intervals are labelled as “gait” when steps are detected; step detector is based on Ryu *et al*.^40^.

### State probability and transitions

Considering the temporal locomotor behaviour to be a discrete time stochastic process *X*_*k*_, *k* ∈ {1, *N*}, where, *N* is the length of the observed time series, the probability (SP) of the process *X* being in a state *j* ∈ {1, *d*} at time *k* is defined as *π*_*j*_(*k*) = Pr(*X*_*k*_ = *j*). *d* is the total number of states in the system. The total probability of the state over the observation period *T* ≤ *N* is thus

1 $${\pi }_{j}=\frac{1}{T}\,\sum _{k=1}^{T}\,{\rm{\Pr }}({X}_{k}=j),\,{\rm{with}}\,\sum _{j=1}^{d}\,{\pi }_{j}=1.$$

This lends to the generalisability of the SP for any time window *T* > 1; at *T* = 1, *π*_*j*_ = 1. Similarly, denoting the probability of transition of the system from a state *i* at time *k* to a state *j* at time *k* + *n* by

2 $${p}_{ij}(n)=\sum _{k}\,{\rm{\Pr }}({X}_{k}=i|{X}_{k+n}=j),\,i,j\in \{1,d\};$$

the transition rate (TR) is then given by

3 $${a}_{ij}(n)={p}_{ij}(n)/\sum _{i}\,{p}_{ij}(n),$$

making the transition matrix **A**_*d*×*d*_ = *a*_*ij*_(*n*) a right stochastic matrix, i.e., $\sum _{i}\,{a}_{ij}=1$. As with the SP, the time window *n* can be varied to suit the objective of the investigation, but we set *n* = 1 in this work. The diagonal elements of the transition matrix represent the probability of being in the same state over the time window *n*.

Thus, considering a *d*–state model for the locomotor behaviour, we have *d* SPs and *d*^2^ TRs which can now be used as features for statistical learning. Noting that the *π*_*j*_ and *a*_*ij*_ are now bounded, the features are now bounded and standardised which makes them amenable for use in various statistical learning algorithms.

Readers familiar with the Markov Models^2^, will recognise tat the SP and TR are the key building blocks of Markov chain models, where the current state of the system can be modelled applying the TR to the SP at the previous time step. However, in this work, we do not attempt to model temporal dynamics of the state of the system, but show that the rich structural information in the TR and the SP (which now, in our case, represent the structure of the system as a whole, over the observation period) can be used to distinguish between various locomotor behaviour.

### Statistical analysis

Each of the feature sets are subjected to standard statistical analysis, in terms of the descriptive statistics, i.e. population mean, standard deviation, skewness, kurtosis, three quartiles (25^th^, 50^th^ or median, and 75^th^ percentiles). The differences in the population density of the two groups are investigated through the Mann-Whitney U test, while the tests for normality are performed through the use of the Shapiro-Francia test (which generalises Shapiro-Wilks test in the presence of skewness).

### Statistical learning

We analyse the two feature sets derived above, namely the SP and TR for supervised classification between different groups representing different collective locomotor behaviour. To this end, we use a number of popular supervised classification algorithms: k-nearest neighbours (k-NN), quadratic discriminant analysis (QDA), Support Vector Machine (SVM), Neural Network (NNet), Naïve Bayes (NB) classifier, and ensemble based methods such as Decision Trees (DT), Adaptive Boosting (AdaBoost) and Random Forests (RF). For the sake of brevity, the methods are not explained here, but the readers are referred to Bishop^2^ and Hastie *et al*.^41^ for details on the algorithms. The supervised learning algorithms were implemented using the open-source machine learning library *scikit*-*learn* in Python.

We perform two different investigations here: (a) compare the performance of the simplified SP as opposed to the more complex TR features; and (b) determine the relevance of each of the locomotor states and associated transitions in distinguishing the distinct locomotor behaviour. We assert that the objective of these two investigations are motivated by the desire to develop parsimonious models for analysing the locomotor behaviour and drawing insights into to dynamics of such behaviour.

#### Validation and performance

The algorithms are trained and tested through a *k*-fold cross validation (CV) scheme, and the performance accuracy is calculated. Since hyper-parameter optimisation for each method is not attempted here (as a proof-of-principle, the algorithms are used in their default settings), this implementation is deemed to be appropriate in this work. When hyper-parameter optimisation is attempted, the data should be split into training and testing sets, with a *k*–fold CV for optimisation performed on the training set, and performance and validation performed on only the testing set. Further analysis of the performance of the algorithms is effected through the Receiver-Operator-Characteristics (ROC) curves, more specifically the area under the ROC curves (referred to as the AUCROC here). This metric is a popular model comparison method that with higher values (the AUCROC is bounded in [0, 1]) suggesting better classification performance. We designate the AUCROC scores of [0.7, 0.8) fair, [0.8, 0.9) good, and [0.9, 1.0] excellent, as performance descriptors in this text.

#### Feature importance

The feature importance in the classification task is evaluated only for the ensemble based methods owing to their ability to provide a one-to-one correspondence between the input variables and the features selected by the algorithms for maximising accuracy. Other methods such as neural networks often transform features in the process, and are not readily interpretable in the context of the input variables. The feature importance here is calculated through the Gini impurity index^42^, defined as follows. If there exist *k* classes, and if *f*_*i*_ are the fraction of elements labelled as *i*, *i* ∈ {1, 2, 3, …, *k*}, then the Gini impurity index, ${I}_{G}=\sum _{i=1}^{k}\,{f}_{i}(1-{f}_{i})$.

### Ethics approval

The experimental protocols were designed in accordance with the relevant guidelines and regulations in the Declaration of Helsinki. Ethical approval for the control study was obtained from the Ethical Committee of the German Sport University Cologne (reference numbers 05/2014 and 38/2015). Ethical approval for the trial at the LVR Hospital, Cologne was obtained from the Ethikkommission der Ärztekammer Nordrhein (Ethics Commission of the Medical Association of North Rhein) with the reference number 2014216, and was registered in the German Clinical Trial Register (DRKS00006740) on October 28, 2014. The trial protocol is outlined in Fleiner *et al*.^25^. Informed consent was obtained from all the subjects and/or their legal guardians.

## References

[1] Willems J (1991). Paradigms and puzzles in the theory of dynamical systems. IEEE Transactions on Automatic Control.

[2] Bishop CM (2006). Pattern Recognition and Machine Learning.

[3] Cuaya G (2013). A dynamic Bayesian network for estimating the risk of falls from real gait data. Med. Biol. Eng. Comput..

[4] Bruijn SM (2010). Estimating dynamic gait stability using data from non-aligned inertial sensors. Annals of Biomedical Engineering.

[5] Palmerini L, Rocchi L, Mellone S, Valzania F, Chiari L (2011). Feature selection for accelerometer-based posture analysis in Parkinsons disease. IEEE Trans. Inf. Technol. Biomed..

[6] Bruijn SM, Meijer OG, Beek PJ, van Dieën JH (2013). Assessing the stability of human locomotion: a review of current measures. J. R. Soc. Interface.

[7] McCrum C (2014). Deficient recovery response and adaptive feedback potential in dynamic gait stability in unilateral peripheral vestibular disorder patients. Physiol. Rep.

[8] Gago MF (2014). Postural Stability Analysis with Inertial Measurement Units in Alzheimer’s Disease E X T R A. Dement Geriatr Cogn Disord Extra.

[9] Hubble RP, Naughton GA, Silburn PA, Cole MH (2015). Wearable Sensor Use for Assessing Standing Balance and Walking Stability in People with Parkinson’s Disease: A Systematic Review. PLoS One.

[10] Bagalà F (2012). Evaluation of Accelerometer-Based Fall Detection Algorithms on Real-World Falls. PLoS One.

[11] Weiss, A., Herman, T., Giladi, N. & Hausdorff, J. M. Objective assessment of fall risk in Parkinson’s disease using a body-fixed sensor worn for 3 days. *PLoS One* **9**, 10.1371/journal.pone.0096675 (2014). 10.1371/journal.pone.0096675 PMC4011791 24801889

[12] Geraedts HAE, Zijlstra W, Van Keeken HG, Zhang W, Stevens M (2015). Validation and user evaluation of a sensor-based method for detecting mobility-related activities in older adults. PLoS One.

[13] Fleiner, T., Haussermann, P., Mellone, S. & Zijlstra, W. Sensor-based assessment of mobility-related behavior in dementia: feasibility and relevance in a hospital context. *Int*. *Psychogeriatrics* 1–8, 10.1017/S1041610216001034 (2016). 10.1017/S1041610216001034 27585706

[14] Zhang W, Regterschot GRH, Geraedts H, Baldus H, Zijlstra W (2017). Chair Rise Peak Power in Daily Life Measured With a Pendant Sensor Associates With Mobility, Limitation in Activities, and Frailty in Old People. IEEE Journal of Biomedical and Health Informatics.

[15] Fleiner T, Dauth H, Gersie M, Zijlstra W, Haussermann P (2017). Structured physical exercise improves neuropsychiatric symptoms in acute dementia care: a hospital-based RCT. Alzheimer’s Research & Therapy.

[16] Hu K (2016). Progression of Dementia Assessed by Temporal Correlations of Physical Activity: Results From a 3.5-Year, Longitudinal Randomized Controlled Trial. Sci. Rep..

[17] Urwyler P (2017). Cognitive impairment categorized in community-dwelling older adults with and without dementia using in-home sensors that recognise activities of daily living. Sci. Rep..

[18] Bonomi AG, Goris AHC, Yin B, Westerterp KR (2009). Detection of type, duration, and intensity of physical activity using an accelerometer. Med. Sci. Sports Exerc..

[19] Mannini A, Sabatini AM (2010). Machine learning methods for classifying human physical activity from on-body accelerometers. Sensors.

[20] Urwyler P (2015). Recognition of activities of daily living in healthy subjects using two ad-hoc classifiers. BioMedical Engineering OnLine.

[21] Yang, J. B., Nguyen, M. N., San, P. P., Li, X. L. & Shonali, K. Deep Convolutional Neural Networks On Multichannel Time Series For Human Activity Recognition. *IJCAI’15 Proceedings of the 24th International Conference on Artificial Intelligence* 3995–4001 (2015).

[22] Lim ASP (2011). Quantification of the fragmentation of rest-activity patterns in elderly individuals using a state transition analysis. Sleep.

[23] Sohail S, Yu L, Bennett DA, Buchman AS, Lim ASP (2015). Irregular 24-hour activity rhythms and the metabolic syndrome in older adults. Chronobiology International.

[24] Draper, B., Finkel, S. I. & Tune, L. An introduction to BPSD. In Draper, B., Brodaty, H. & Finkel, S. I. (eds) *IPA Complet*. *Guid*. *to Behav*. *Psychol*. *Symptoms Dement*. *Spec*. *Guid*., chap. Module I, 1.1–1.13 (International Psychogeriatric Association (IPA), Milwaukee, WI, 2015).

[25] Fleiner T, Zijlstra W, Dauth H, Haussermann P (2015). Evaluation of a hospital-based day-structuring exercise programme on exacerbated behavioural and psychological symptoms in dementia - the exercise carrousel: study protocol for a randomised controlled trial. Trials.

[26] Bourke A (2017). A Physical Activity Reference Data-Set Recorded from Older Adults Using Body-Worn Inertial Sensors and Video Technology—The ADAPT Study Data-Set. Sensors.

[27] Breiman L (2001). Random Forests. Machine Learning.

[28] Menze BH (2009). A comparison of random forest and its Gini importance with standard chemometric methods for the feature selection and classification of spectral data. BMC Bioinformatics.

[29] Van Alphen HJM (2016). Older adults with dementia are sedentary for most of the day. PLoS One.

[30] Cummings JL (1994). The Neuropsychiatric Inventory: Comprehensive assessment of psychopathology in dementia. Neurology.

[31] Giannouli E, Bock O, Mellone S, Zijlstra W (2016). Mobility in Old Age: Capacity Is Not Performance. Biomed Res. Int..

[32] World Health Organization. *The ICD*-*10 Classification of Mental and Behavioural Disorders*: *Diagnostic Criteria for Research*. ICD-10 classification of mental and behavioural disorders/World Health Organization (World Health Organization, 1993).

[33] Mellone S, Tacconi C, Chiari L (2012). Validity of a Smartphone-based instrumented Timed Up and Go. Gait Posture.

[34] Maetzler W, Domingos J, Srulijes K, Ferreira JJ, Bloem BR (2013). Quantitative wearable sensors for objective assessment of Parkinson’s disease. Mov. Disord..

[35] Chigateri, N., Kerse, N., MacDonald, B. & Klenk, J. Validation of Walking Episode Recognition in Supervised and Free-Living Conditions Using Triaxial Accelerometers. In *Proc*. *2017 World Congr*. *Int*. *Soeciety Posture Gait Res*., 289–290 (Florida, USA, 2017).

[36] Leach JM, Mellone S, Palumbo P, Bandinelli S, Chiari L (2018). Natural turn measures predict recurrent falls in community-dwelling older adults: a longitudinal cohort study. Sci. Rep..

[37] Mansoubi M (2015). Energy expenditure during common sitting and standing tasks: examining the 1.5 MET definition of sedentary behaviour. BMC Public Health.

[38] Sasaki JE, John D, Freedson PS (2011). Validation and comparison of ActiGraph activity monitors. J. Sci. Med. Sport.

[39] Zijlstra A, Mancini M, Lindermann U, Chiari L, Zijlstra W (2012). Sit- stand and stand-sit transitions in older adults and patients with Parkinson’s disease: event detection based on motion sensors versus force plates. J. Neuroeng. Rehabil..

[40] Ryu, U. *et al*. Adaptive Step Detection Algorithm for Wireless Smart Step Counter. In *2013 International Conference on Information Science and Applications* (*ICISA*), 1–4, 10.1109/ICISA.2013.6579332 (IEEE, 2013).

[41] Hastie, T., Tibshirani, R. & Friedman, J. *The Elements of Statistical Learning*. Springer Series in Statistics (Springer New York, New York, NY, 2009).

[42] Gras, R. & Kuntz, P. Reduction of Redundant Rules in Statistical Implicative Analysis. In Brito, P., Cucumel, G., Bertrand, P. & de Carvalho, F. (eds) *Sel*. *Contrib*. *Data Anal*. *Classif*., 367–376, 10.1007/978-3-540-73560-1_34 (Springer Berlin, Heidelberg, 2007).

[43] Cramer, D. *Basic statistics for social research* (Routledge, Oxford, 1997).

